# The COVID-19 Pandemic and Adolescents’ Experience in Sub-Saharan Africa: A Cross-Country Study Using a Telephone Survey

**DOI:** 10.4269/ajtmh.20-1620

**Published:** 2021-06-23

**Authors:** Dongqing Wang, Angela Chukwu, Ourohiré Millogo, Nega Assefa, Christabel James, Tara Young, Bruno Lankoande, Firehiwot Workneh, Elena C. Hemler, Michelle L. Korte, Josiemer Mattei, Abdramane Bassiahi Soura, Ali Sie, Ayoade Oduola, Yemane Berhane, Wafaie W. Fawzi

**Affiliations:** 1 Department of Global Health and Population, Harvard T.H. Chan School of Public Health, Harvard University, Boston, Massachusetts;; 2 Department of Statistics, University of Ibadan, Ibadan, Nigeria;; 3 Nouna Health Research Center, Nouna, Burkina Faso;; 4 College of Health and Medical Sciences, Haramaya University, Harar, Ethiopia;; 5 Institut Supérieur des Sciences de la Population, University of Ouagadougou, Ouagadougou, Burkina Faso;; 6 Addis Continental Institute of Public Health, Addis Ababa, Ethiopia;; 7 Department of Nutrition, Harvard T.H. Chan School of Public Health, Harvard University, Boston, Massachusetts;; 8 University of Ibadan Research Foundation, University of Ibadan, Ibadan, Nigeria;; 9 Department of Epidemiology, Harvard T.H. Chan School of Public Health, Harvard University, Boston, Massachusetts

## Abstract

The public health measures instituted by governments to combat the coronavirus disease 2019 (COVID-19) may cause developmental and educational losses to adolescents. The impacts of the COVID-19 pandemic and its mitigation strategies on adolescents in sub-Saharan Africa are unclear. This study aimed to examine adolescents’ knowledge, perceptions, and practices related to COVID-19 and the impacts of the pandemic on the daily lives of adolescents in sub-Saharan Africa. The survey was conducted in Burkina Faso, Ethiopia, and Nigeria using computer-assisted telephone interviews to enable rapid and remote data collection. Two sites were included in each country, with approximately 300 adolescents per site and 1,795 adolescents in total. Variations across the six sites were noted for the proportions of the adolescents who could correctly identify all key COVID-19 symptoms (4–25%), transmission methods (16–59%), and prevention approaches (33–79%). Most (> 72%) of the adolescents were no longer going to school due to school closures. Many adolescents (23–81%) were not receiving any education during the pandemic. A considerable proportion of the adolescents (44–83%) self-assessed as having less ability to learn during the pandemic; many expected it to be very difficult to catch up on education after the pandemic. Decreases in the consumption of major food groups were common across sites. Urgent actions are needed in sub-Saharan Africa to address the inadequate knowledge of COVID-19 among adolescents and the impacts of the pandemic on adolescent education and nutrition.

## INTRODUCTION

The coronavirus disease 2019 (COVID-19), caused by severe acute respiratory syndrome coronavirus 2 (SARS-CoV-2), creates unprecedented challenges worldwide, including in sub-Saharan Africa (SSA). To respond to the COVID-19 pandemic, many countries in SSA have instituted public health measures at national levels, such as stay-at-home orders and physical distancing requirements, to minimize the spread of the disease and reduce the burden on healthcare systems.^1^ These mitigation measures have persisted to some level even to this date. For example, as of February 2021, school closures were still recommended in Ethiopia and required at all levels in Nigeria; stay-at-home restrictions (except for essentials) were still in place in most countries in SSA.^1^

Fewer deaths from the COVID-19 disease have been reported in SSA than the rest of the world,^2^ which may be attributable to the delayed arrival of the virus in SSA,^3^ the generally younger population distribution of the region,^4^ and Africa’s quick actions to prepare and respond to this crisis.^5^ Compared with elderly individuals and adults with comorbidities, the virus appears to cause mild or no symptoms, less severe disease outcomes, and lower case‐fatality rates among adolescents aged 10–19 years, a generally healthy group.^6^ Adolescence is a unique transitional life period between childhood and adulthood, marked by crucial physical, mental, and social developments.^7^ Lifestyle behaviors established in adolescence, such as dietary intake and physical activity, create patterns that shape behaviors through adulthood and can have long-term effects on health outcomes.^8,9^

With a nominal rate of case fatality among this age group,^10^ adolescents do not directly suffer from a high burden of COVID-19. However, the public health measures put in place to combat the pandemic, such as extended self-isolation and physical distancing, lockdowns of communities, and the closures of schools, may have adverse collateral effects on the development and health of adolescents that may be difficult to reverse.^11–13^ In addition to the direct losses of educational opportunities due to school closures, school-based nutrition programs, such as school feeding and nutrition education, are also disrupted by the pandemic. A recent cross-sectional study in Kampala, Uganda, reports that COVID-19 lockdowns led to mental health challenges, reduced ability to meet basic needs, disruptions to socioeconomic status, and engagement in unhealthy behaviors among adolescent boys and young men.^14^ However, the short- and long-term impacts of the COVID-19 pandemic and its mitigation strategies on adolescents girls and boys from diverse settings in countries in SSA are still poorly understood, especially regarding the effects on education and nutrition.

Adolescence is critical for the health of the world population, both through direct impacts on development and indirect impacts on health and well-being during later life stages.^7^ Understanding the impacts of the COVID‐19 pandemic on adolescents is important to the design and targeting of interventions during and after the pandemic, especially in SSA, where adolescents are often overlooked in public programming. We aimed to use data from a multi-country, phone-based survey to examine adolescents’ knowledge and perceptions of COVID-19, the use of COVID-19 preventive strategies among adolescents, and the impacts of the COVID-19 pandemic and its mitigation strategies on various aspects of the adolescents’ lives in SSA.

## MATERIALS AND METHODS

### Study design and study population.

This study is based on an ongoing phone-based longitudinal survey that uses a novel mobile platform and computer-assisted telephone interviewing (CATI) to collect data in three countries in SSA: Ethiopia, Burkina Faso, and Nigeria. The first cases of COVID-19 were reported in late February in Nigeria and in March in Burkina Faso and Ethiopia. In each country, we included one rural site and one urban site: Nouna (rural) and Ouagadougou (urban) in Burkina Faso, Kersa (rural) and Addis Ababa (urban) in Ethiopia, and Ibadan (rural) and Lagos (urban) in Nigeria. The study rationale, sampling strategies, the use of CATI technology, and the detailed study instruments are described in detail elsewhere.^15^ Briefly, households were selected from sampling frames of existing Health and Demographic Surveillance Systems or national surveys, where possible. Sites used different household sampling frames depending on the platforms available, including 1) existing Health and Demographic Surveillance Systems in Burkina Faso and rural Ethiopia (Kersa), 2) National Living Standard Survey and an existing adolescent study in Nigeria, and 3) a new household survey in urban Ethiopia (Addis Ababa). Within each sampling frame, households were randomly selected, and we contacted approximately 300 households in each site that have adolescents between the ages of 10–19 years. One adolescent was interviewed in each household; when two or more adolescents were present in a household, one with the next birthday as of the date of the interview was selected to ensure a random selection within a household. Between July and November 2020, trained research staff conducted the survey in the local language of each site.

Verbal parental consent and adolescent assent were obtained for adolescents younger than 18 years of age; verbal informed consent was obtained from adolescents aged 18 years of age and older. Specifically, a data collector called the adult of each household to describe the purpose of the study and obtain verbal informed consent using a standardized consent script. If the participant was a parent or guardian of an adolescent in the household, the data collector also sought verbal informed consent to interview the adolescent. If parental informed consent for adolescent interview was granted, the data collector then sought verbal informed assent from the adolescent using a standardized assent script. All research staff was trained on study procedures, including screening, consent, enrollment, and data collection, with an emphasis on safeguarding the privacy and confidentiality of study participants during remote interviews. The surveys were administered by staff trained in the protection of participant privacy and confidentiality. All participants were instructed to receive the interview in a private space away from other people, if possible.

This study was approved by the Institutional Review Board at Harvard T.H. Chan School of Public Health and ethical review boards in each country and site, including Nouna Health Research Center Ethical Committee and National Ethics Committee in Burkina Faso, the Institutional Ethical Review Board of Addis Continental Institute of Public Health in Ethiopia, and the University of Ibadan Research Ethics Committee and National Health Research Ethics Committee in Nigeria.

### Data collection.

The survey collected data on adolescent sociodemographic characteristics (age, sex, education level, and occupation) and assessed adolescents’ knowledge on 1) COVID-19 symptoms, 2) COVID-19 transmission methods, and 3) COVID-19 prevention methods. The survey also collected information on adolescents’ perceptions of COVID-19 and their use of COVID-19 preventive strategies. Finally, the survey inquired about the impacts of the COVID-19 pandemic on adolescents’ education, daily activities, communication, media consumption, and various health domains, including dietary intake and mental health. Data collectors entered participant responses directly into a mobile tablet-based data collection system, and data were uploaded to a secure server for statistical analysis. The adolescent questionnaire was developed by working groups comprised of subject matter experts from participating institutions in the ARISE network^16^ based on existing resources.^17–19^ A small pilot study using the questionnaire was conducted in each country to allow for feedback and minor modifications prior to the actual data collection.

### Statistical analysis.

We calculated means and SDs overall and by site for normally distributed continuous variables; medians, 25th percentiles (Q1), and 75th percentiles (Q3) for skewed continuous variables; and counts and percentages for categorical variables. For each of the three domains of COVID-19 knowledge (symptoms, transmission, and prevention), we created a score based on the number of correct responses. A correct response was given one point; incorrect response, “don’t know,” refusal to answer, and missing response were all given zero points. A higher score indicated greater knowledge in that domain. The total scores for symptoms, transmission, and prevention were 10, 5, and 7, respectively, with 0 being the theoretical minimum score for each; the cutoffs to be considered as having reasonably good knowledge were 8, 4, and 6, respectively. In addition to the domain-specific scores, we added up the three scores to construct a total knowledge score of COVID-19 with a theoretical range of 0–22; the cutoff for having reasonably good knowledge based on the total score was set at 18. All the aforementioned cutoffs for knowledge scores were determined to correspond to an 80% correctness.

Mental health was measured using the four-item Patient Health Questionnaire for Depression and Anxiety Scale (PHQ-4),^17^ which included four questions related to psychological distress: “Feeling nervous, anxious or on edge,” “Not being able to stop or control worrying,” “Little interest or pleasure in doing things,” and “Feeling down, depressed, or hopeless.” Each question has four possible responses, including “Not at all,” “Several days,” “More than half the days,” and “Nearly every day” over the past 2 weeks, corresponding to a numeric score of 0, 1, 2, and 3, respectively. A total score for psychological distress was computed by adding up the scores of the four items, with a range of 0–12. We further categorized psychological distress as none (total score: 0–2), mild (total score: 3–5), moderate (total score: 6–8), and severe (total score: 9–12).^17^ We also created an anxiety subscale (range: 0–6) using the scores of the first two questions and a depression subscale (range: 0–6) using the scores of the last two questions. A subscale score of 3 or greater was considered a high level of anxiety or depression, respectively.^17^ The PHQ-4 has previously been shown to be valid and reliable for screening anxiety and depression among adolescents in SSA.^20^ We conducted all analyses using SAS 9.4 (SAS Institute Inc., Cary, NC) at a two-sided alpha level of 0.05.

## RESULTS

### Sociodemographic characteristics.

A total of 1,795 adolescents were interviewed, with approximately 300 in each site (Table 1). The age of the adolescents ranged from 10 years to 19 years. The median age was 15 (Ouagadougou, Kersa, and Ibadan), 16 (Nouna), or 17 (Addis Ababa and Lagos) years. The sex distributions were roughly balanced, with approximately the same number of girls versus boys, except for Kersa (32% were girls) and Addis Ababa (66% were girls). The majority (> 95%) of the adolescents had at least some primary school education, except in Nouna, where 20% of the participants did not have any primary school education. The majority (> 95%) of the adolescents were students, except in the two sites of Burkina Faso, where around half of the participants were students.

**Table 1:** Sociodemographic characteristics of adolescents in a phone-based survey on the impacts of the COVID-19 pandemic on adolescent experience across six sites of three sub-Saharan African countries, 2020*

Setting	Burkina Faso		Ethiopia		Nigeria		Total
	Rural	Urban	Rural	Urban	Rural	Urban	
	Nouna	Ouagadougou	Kersa	Addis Ababa	Ibadan	Lagos	
Number of adolescents, *N*	297	300	294	296	365	243	1,795
Age, years	16 (14, 18)	15 (13, 17)	15 (12, 18)	17 (15, 18)	15 (13, 17)	17 (14, 19)	16 (13, 18)
Girls, *N* (%)	140 (47.1)	161 (53.7)	94 (32.0)	194 (65.5)	217 (59.5)	129 (53.1)	935 (52.1)
Highest level of education, *N* (%)							
None/religious school/literacy class	60 (20.2)	13 (4.33)	6 (2.04)	3 (1.01)	0 (0)	2 (0.82)	84 (4.68)
Some primary school	81 (27.3)	69 (23.0)	212 (72.1)	101 (34.1)	11 (3.01)	3 (1.23)	477 (26.6)
Completed primary school	40 (13.5)	49 (16.3)	19 (6.46)	41 (13.9)	102 (28.0)	42 (17.3)	293 (16.3)
Some secondary/high school	107 (36.0)	157 (52.3)	47 (16.0)	85 (28.7)	221 (60.6)	118 (48.6)	735 (41.0)
Completed secondary/high school	7 (2.36)	12 (4.00)	5 (1.70)	41 (13.9)	26 (7.12)	59 (24.3)	150 (8.36)
Tertiary education or higher	2 (0.67)	0 (0)	5 (1.70)	25 (8.45)	5 (1.37)	19 (7.82)	56 (3.12)
Occupation, *N* (%)†							
Unemployed	9 (3.03)	59 (19.7)	8 (2.72)	8 (2.70)	2 (0.55)	1 (0.41)	87 (4.85)
Student	149 (50.2)	133 (44.3)	281 (95.6)	285 (96.3)	359 (98.4)	235 (96.7)	1,442 (80.3)
Farmer	100 (33.7)	0 (0)	23 (7.82)	0 (0)	1 (0.27)	0 (0)	124 (6.91)
Wage employment	0 (0)	2 (0.67)	1 (0.34)	2 (0.68)	3 (0.82)	4 (1.65)	12 (0.67)
Self-used	4 (1.35)	19 (6.33)	2 (0.68)	3 (1.01)	2 (0.55)	4 (1.65)	34 (1.89)
Other	19 (6.40)	89 (29.7)	1 (0.34)	1 (0.34)	7 (1.92)	4 (1.65)	121 (6.74)

* Values are median (25th percentile, 75th percentile) for skewed continuous variables and count (percentage) for categorical variables.

† Counts and percentages do not add up to the total because the selection of multiple responses was allowed.

### Knowledge, perceptions, and practices related to COVID-19.

The proportion of the interviewed adolescents who did not believe COVID-19 was real ranged from 2% in Ouagadougou to 9% in Nouna (Table 2). The proportion of the adolescents who were not concerned about the spread of COVID-19 ranged from 12% in Ouagadougou and Kersa to 28% in Addis Ababa. In Burkina Faso and Ethiopia, 56–89% of adolescents perceived themselves to be at no risk or low risk of exposure to COVID-19 (Supplemental Figure 1); this proportion was slightly lower in the two sites of Nigeria (43% in Ibadan and 42% in Lagos). Although a large proportion of the adolescents had reasonably good knowledge (i.e., knowledge score ≥ 8 out of a max of 10) of COVID-19 symptoms, few could correctly identify all key symptoms of COVID-19, ranging from 4% in Kersa to 25% in Ouagadougou. Knowledge of COVID-19 transmission methods and prevention approaches was comparatively better, with the proportions of adolescents who could correctly identify all transmission methods ranging from 16% in Nouna to 59% in Lagos and who could correctly identify all prevention approaches ranging from 33% in Nouna to 79% in Addis Ababa. Few adolescents (ranging from 1.4% in Nouna and Kersa to 9% in Ouagadougou) could identify all COVID-19 symptoms, transmission methods, and prevention approaches (Supplemental Figure 2). Primary sources of information on COVID-19 for adolescents were radio (> 70% in all sites except Addis Ababa), television (> 60% in all sites except Kersa), and friends or family (ranging from 26% in Kersa to 58% in Ouagadougou). Government messages were more common in Addis Ababa (36%) and both sites in Nigeria (32% in Ibadan and 51% in Lagos). COVID-19 prevention measures were used by most adolescents, including regular handwashing (ranging from 85% in Kersa to 98% in Ouagadougou and Addis Ababa) and wearing face masks (ranging from 63% in Nouna to 95% in Ouagadougou). Disinfecting surfaces and using physical distancing strategies were also commonly adopted across sites (Table 2).

**Table 2:** Knowledge, perceptions, and practices related to COVID-19 among adolescents in a phone-based survey across six sites of three sub-Saharan African countries, 2020*

Setting	Burkina Faso		Ethiopia		Nigeria		Total
	Rural	Urban	Rural	Urban	Rural	Urban	
	Nouna	Ouagadougou	Kersa	Addis Ababa	Ibadan	Lagos	
Number of adolescents, *N*	297	300	294	296	365	243	1,795
Believe COVID-19 is real,† *N* (%)							
Yes	262 (88.2)	294 (98.0)	271 (92.2)	273 (92.2)	346 (95.1)	232 (95.5)	1,678 (93.5)
No	26 (8.75)	6 (2.00)	21 (7.14)	15 (5.07)	16 (4.40)	8 (3.29)	92 (5.13)
Not sure	9 (3.03)	0 (0)	2 (0.68)	8 (2.70)	2 (0.55)	3 (1.23)	24 (1.34)
Not concerned about the spread of COVID-19,‡ *N* (%)	47 (16.9)	35 (11.7)	35 (12.2)	83 (28.0)	78 (21.5)	37 (15.6)	315 (17.9)
Knowledge score of COVID-19 symptoms (theoretical range: 0–10)	8 (6, 9)	9 (8, 9)	8 (7, 9)	8 (7, 9)	7 (5, 9)	8 (6, 9)	8 (6, 9)
Knowledge score of COVID-19 transmission methods (theoretical range: 0–5)	4 (3, 4)	4 (4, 5)	4 (4, 4)	4 (4, 5)	5 (4, 5)	5 (4, 5)	4 (4, 5)
Knowledge score of COVID-19 prevention methods (theoretical range: 0–7)	6 (6, 7)	7 (6, 7)	7 (6, 7)	7 (7, 7)	6 (6, 7)	7 (6, 7)	7 (6, 7)
Total knowledge score of COVID-19 (theoretical range: 0–22)	18 (15, 19)	20 (18, 21)	19 (17, 20)	19 (18, 20)	18 (15, 19)	18 (17, 20)	19 (17, 20)
Sources of information on COVID-19,§‖ *N* (%)							
Friends/family	158 (53.4)	174 (58.0)	75 (25.5)	87 (29.4)	113 (31.0)	114 (47.1)	721 (40.2)
Government messages	43 (14.5)	23 (7.67)	33 (11.2)	107 (36.2)	116 (31.8)	123 (50.8)	445 (24.8)
Newspapers	22 (7.43)	10 (3.33)	1 (0.34)	5 (1.69)	100 (27.4)	123 (50.8)	261 (14.6)
Radio	243 (82.1)	222 (74.0)	256 (87.1)	99 (33.5)	275 (75.3)	186 (76.9)	1,281 (71.4)
Social media	26 (8.78)	62 (20.7)	25 (8.50)	59 (19.9)	172 (47.1)	172 (71.1)	516 (28.8)
Television	238 (80.4)	237 (79.0)	97 (33.0)	280 (94.6)	221 (60.6)	191 (78.9)	1,264 (70.5)
Internet search	5 (1.69)	9 (3.00)	6 (2.04)	81 (27.4)	72 (19.7)	81 (33.5)	254 (14.2)
Not receiving information	1 (0.34)	2 (0.67)	1 (0.34)	0 (0)	0 (0)	0 (0)	4 (0.22)
Other	6 (2.03)	12 (4.00)	0 (0)	13 (4.39)	1 (0.27)	4 (1.65)	36 (2.01)
Measures taken to respond to the COVID-19 pandemic since the beginning,§¶ *N* (%)							
Nothing	2 (0.68)	2 (0.67)	10 (3.40)	2 (0.68)	2 (0.55)	3 (1.23)	21 (1.17)
Regular handwashing with soap and water	286 (96.6)	295 (98.3)	250 (85.0)	289 (97.6)	342 (93.7)	222 (91.4)	1,684 (93.9)
Disinfecting surfaces	49 (16.6)	60 (20.0)	8 (2.72)	195 (65.9)	225 (61.6)	156 (64.2)	693 (38.6)
Keeping distance from sick people	194 (65.5)	166 (55.3)	91 (31.0)	147 (49.7)	254 (69.6)	171 (70.4)	1,023 (57.0)
Keeping distance from people outside of the household	105 (35.5)	177 (59.0)	203 (69.1)	219 (74.0)	210 (57.5)	165 (67.9)	1,079 (60.1)
Stopping going to social gatherings	102 (34.5)	49 (16.3)	131 (44.6)	192 (64.9)	152 (41.6)	126 (51.9)	752 (41.9)
Wearing a face mask	187 (63.2)	284 (94.7)	240 (81.6)	257 (86.8)	299 (81.9)	196 (80.7)	1,463 (81.6)
Stocking up on food, home supplies, and medicine	1 (0.34)	7 (2.33)	7 (2.38)	57 (19.3)	62 (17.0)	53 (21.8)	187 (10.4)
Changing/canceling travel plans	52 (17.6)	17 (5.67)	4 (1.36)	89 (30.1)	104 (28.5)	67 (27.6)	333 (18.6)
Other	3 (1.01)	14 (4.67)	15 (5.10)	16 (5.41)	0 (0)	9 (3.70)	57 (3.18)

* Values are median (25th percentile, 75th percentile) for skewed continuous variables and count (percentage) for categorical variables.

† Information was missing for one participant in Ibadan.

‡ Information was missing for 19 participants in Nouna, six participants in Kersa, two participants in Ibadan, and five participants in Lagos.

§ Counts and percentages do not add up to the total because the selection of multiple responses was allowed.

‖ Information was missing for one participant in Nouna and one participant in Lagos.

¶ Information was missing for one participant in Nouna.

### Impacts of the COVID-19 pandemic on daily activities, physical activity, and education.

Most of the interviewed adolescents reported some impacts of the COVID-19 pandemic on their daily lives (Table 3). Across sites, 21% (in Nouna) to 87% (in Ibadan) of adolescents were staying home more often. The proportion of adolescents who experienced a decrease in physical activity during the COVID-19 pandemic compared with before the pandemic ranged from 12% in Ibadan and Lagos to 45% in Kersa.

**Table 3:** Impacts of the COVID-19 pandemic on daily activities, physical activity, and education among the adolescents in a phone-based survey across six sites of three sub-Saharan African countries, 2020*

Setting	Burkina Faso		Ethiopia		Nigeria		Total
	Rural	Urban	Rural	Urban	Rural	Urban	
	Nouna	Ouagadougou	Kersa	Addis Ababa	Ibadan	Lagos	
Number of adolescents, *N*	297	300	294	296	365	243	1,795
Impacts of the COVID-19 pandemic on daily activities,†‡ *N* (%)							
No impacts	48 (16.4)	6 (2.00)	8 (2.73)	16 (5.42)	3 (0.82)	4 (1.67)	85 (4.76)
No longer going to school	211 (72.0)	263 (87.7)	272 (92.8)	257 (87.1)	335 (91.8)	207 (86.3)	1,545 (86.5)
No longer earning money	48 (16.4)	88 (29.3)	68 (23.2)	52 (17.6)	191 (52.3)	135 (56.3)	582 (32.6)
Staying at home more often	61 (20.8)	192 (64.0)	118 (40.3)	231 (78.3)	318 (87.1)	182 (75.8)	1,102 (61.7)
Increased responsibilities at home	29 (9.90)	34 (11.3)	12 (4.10)	88 (29.8)	248 (68.0)	131 (54.6)	542 (30.4)
Gaining weight	18 (6.14)	28 (9.33)	10 (3.41)	66 (22.4)	191 (52.3)	141 (58.8)	454 (25.4)
Losing weight	20 (6.83)	41 (13.7)	40 (13.7)	20 (6.78)	28 (7.67)	33 (13.8)	182 (10.2)
Physical activity before the COVID-19 pandemic,§ *N* (%)							
None	105 (35.7)	47 (15.7)	110 (37.5)	74 (25.0)	44 (12.3)	40 (16.8)	420 (23.6)
< 3 hours/week	159 (54.1)	134 (44.7)	92 (31.4)	126 (42.6)	238 (66.3)	125 (52.5)	874 (49.1)
3–6 hours/week	23 (7.82)	84 (28.0)	70 (23.9)	53 (17.2)	62 (17.3)	41 (17.2)	331 (18.6)
7–9 hours/week	6 (2.04)	20 (6.67)	19 (6.48)	31 (10.5)	9 (2.51)	20 (8.40)	105 (5.90)
10 hours or more	1 (0.34)	15 (5.00)	2 (0.68)	14 (4.73)	6 (1.67)	12 (5.04)	50 (2.81)
Physical activity in the past one week,‖ *N* (%)							
None	158 (53.7)	133 (44.3)	222 (76.0)	137 (46.3)	58 (16.2)	44 (18.5)	752 (42.3)
< 3 hours/week	116 (39.5)	99 (33.0)	41 (14.0)	94 (31.8)	208 (58.1)	122 (51.3)	680 (38.3)
3–6 hours/week	14 (4.76)	38 (12.7)	26 (8.90)	31 (10.5)	61 (17.0)	41 (17.2)	211 (11.9)
7–9 hours/week	6 (2.04)	18 (6.00)	2 (0.68)	27 (9.12)	19 (5.31)	19 (7.98)	91 (5.12)
10 hours or more	0 (0)	12 (4.00)	1 (0.34)	7 (2.36)	12 (3.35)	12 (5.04)	44 (2.47)
Change in physical activity,¶ *N* (%)							
No change	230 (78.5)	174 (58.0)	150 (51.4)	131 (44.3)	261 (73.5)	177 (74.7)	1,123 (63.3)
Decreased	58 (19.8)	114 (38.0)	131 (44.9)	115 (38.9)	41 (11.6)	29 (12.2)	488 (27.5)
Increased	5 (1.71)	12 (4.00)	11 (3.77)	50 (16.9)	53 (14.9)	31 (13.1)	162 (9.14)
Enrolled in school before the COVID-19 pandemic,# *N* (%)	185 (64.2)	260 (86.7)	275 (93.5)	268 (90.5)	335 (93.8)	205 (85.8)	1,528 (86.1)
School currently closed in response to the COVID-19 pandemic,**†† *N* (%)	111 (61.3)	248 (95.8)	271 (99.3)	259 (96.6)	186 (58.1)	136 (67.7)	1,211 (80.6)
Receiving school meals on one or more school days before the COVID-19 pandemic,**‡‡ *N* (%)	84 (45.4)	69 (26.5)	20 (7.75)	87 (32.8)	27 (8.21)	19 (9.64)	306 (20.5)

* Values are count (percentage).

† Counts and percentages do not add up to the total as the selection of multiple responses was allowed.

‡ Information was missing for four participants in Nouna, one participant in Kersa, one participant in Addis Ababa, and three participants in Lagos.

§ Information was missing for three participants in Nouna, one participant in Kersa, six participants in Ibadan, and five participants in Lagos.

‖ Information was missing for three participants in Nouna, two participants in Kersa, seven participants in Ibadan, and five participants in Lagos.

¶ Information was missing for four participants in Nouna, two participants in Kersa, 10 participants in Ibadan, and six participants in Lagos.

# Information was missing for nine participants in Nouna, eight participants in Ibadan, and four participants in Lagos.

** Among adolescents enrolled in school before the COVID-19 pandemic.

†† Information was missing for four participants in Nouna, one participant in Ouagadougou, two participants in Kersa, 15 participants in Ibadan, and four participants in Lagos.

‡‡ Information was missing for three participants in Addis Ababa, 17 participants in Kersa, six participants in Ibadan, and eight participants in Lagos.

**Figure 1.**
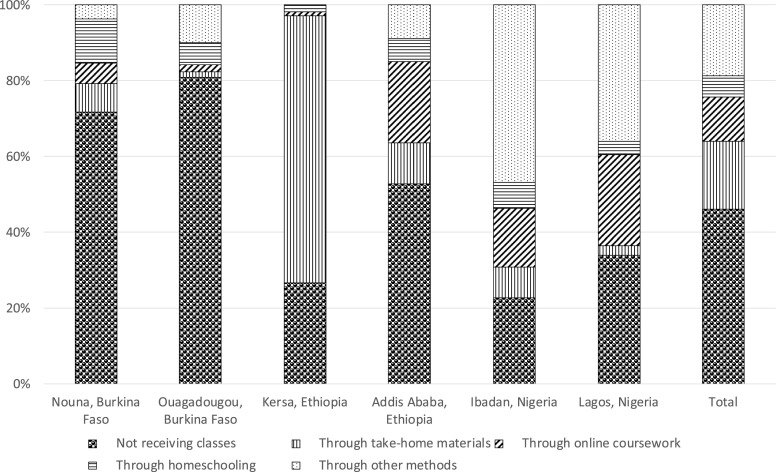
Modes of receiving education among adolescents in a phone-based survey across six sites of three sub-Saharan African countries, 2020. Restricted to adolescents enrolled in school before the COVID-19 pandemic. Information was missing for two participants in Nouna, one participant in Kersa, four participants in Ibadan, and five participants in Lagos.

**Figure 2.**
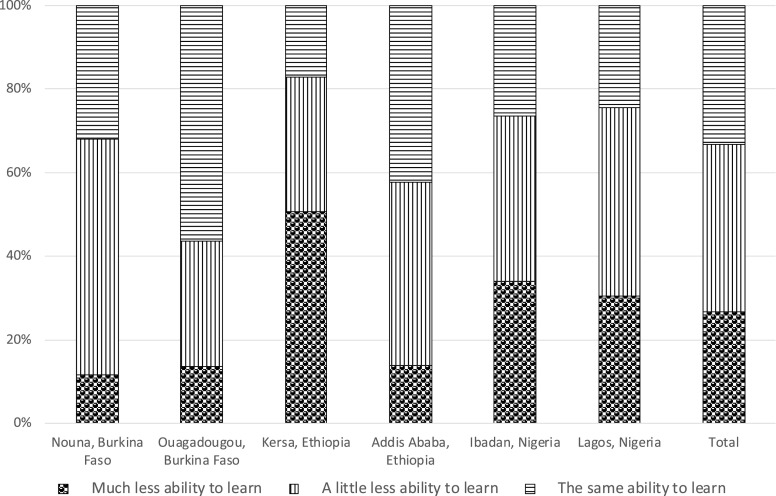
Self-assessed ability to learn during the COVID-19 pandemic compared with before the pandemic among adolescents in a phone-based survey across six sites of three sub-Saharan African countries, 2020. Restricted to adolescents enrolled in school before the COVID-19 pandemic. Information was missing for four participants in Nouna, five participants in Ouagadougou, four participants in Addis Ababa, 12 participants in Kersa, 11 participants in Ibadan, and 12 participants in Lagos.

**Figure 3.**
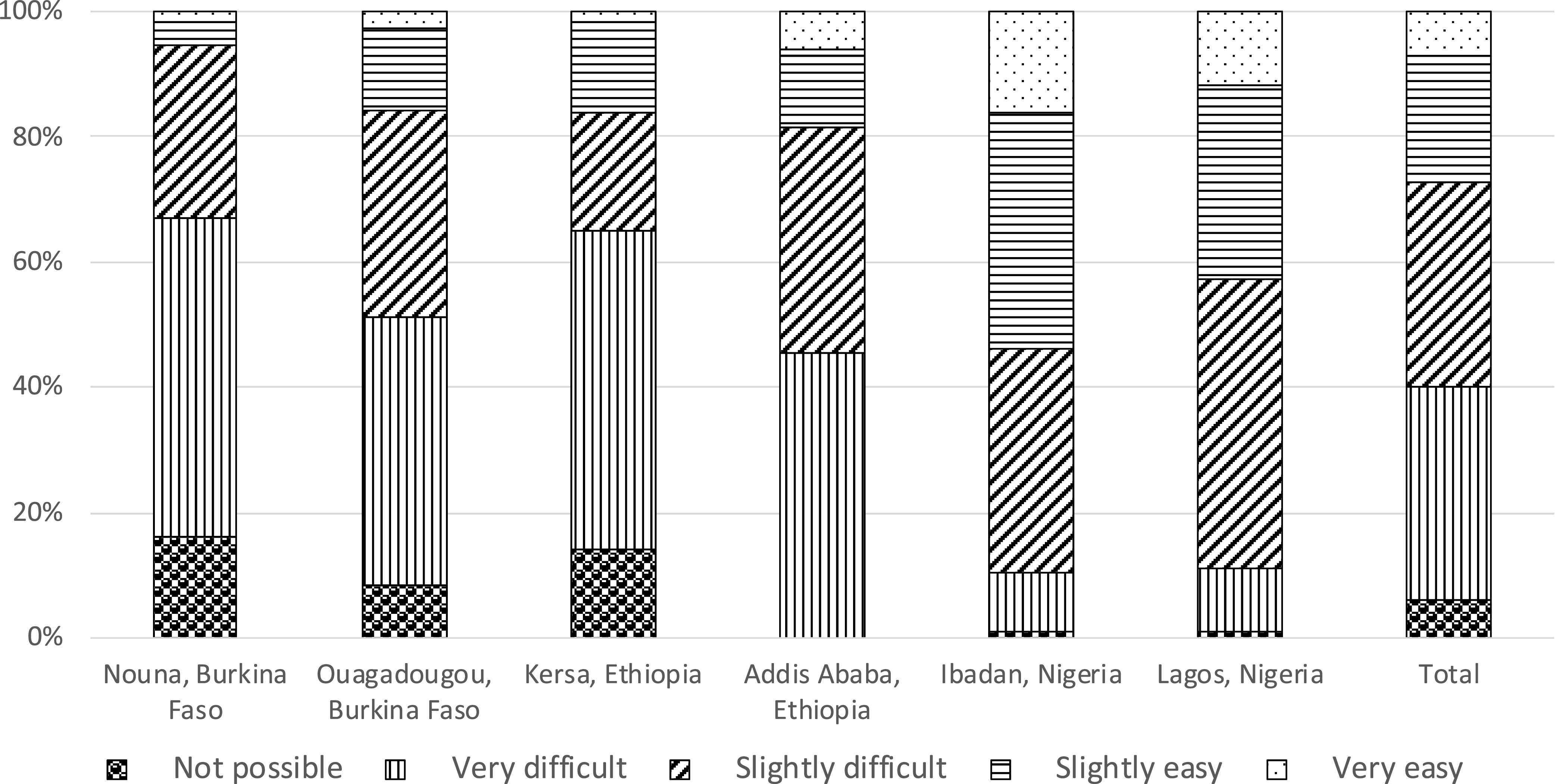
Perceived difficulty of catching up on education after the COVID-19 pandemic among adolescents in a phone-based survey across six sites of three sub-Saharan African countries, 2020. Restricted to adolescents enrolled in school before the COVID-19 pandemic. Information was missing for seven participants in Ouagadougou, seven participants in Addis Ababa, 34 participants in Kersa, eight participants in Ibadan, and eight participants in Lagos.

Among adolescents who were enrolled in school, most reported school closures in response to the pandemic (> 95% in Ouagadougou and both sites in Ethiopia and ∼60% in Nouna, Ibadan, and Lagos; Table 3). In Burkina Faso, over 70% of the adolescents (72% in Nouna and 81% in Ouagadougou) were not receiving any education at all during the pandemic. In Kersa, more than 70% received education through take-home materials, and 27% were not receiving any education. In Addis Ababa, 22% and 11% received education through online coursework and take-home materials, respectively, whereas 53% were not receiving any education. In Ibadan, 15% received online coursework, 47% received education from other channels, and 23% did not receive any education. In Lagos, 24% received online coursework, 36% received education from other channels, and 34% did not receive any education (Figure 1). Most adolescents self-assessed as having less ability to learn during the COVID-19 pandemic, ranging from 44% in Ouagadougou to 83% in Kersa (Figure 2). In Burkina Faso and Ethiopia, around half of the adolescents (ranging from 43% in Ouagadougou to 51% in Nouna and Kersa) perceived that it would be *very* difficult to catch up on their education after the pandemic; in Nigeria, 36% in Ibadan and 46% in Lagos perceived it to be *slightly* difficult to catch up on their education (Figure 3).

### Impacts of the COVID-19 pandemic on communication and media consumption.

An appreciable proportion (ranging from 29% in Nouna to 66% in Kersa) of the adolescents reported that the frequency of communicating with family and friends outside their household decreased a lot during the pandemic compared with before the pandemic (Supplemental Figure 3). Phone calls (ranging from 32% in Nouna to 83% in Addis Ababa) and in-person talking (ranging from 27% in Addis Ababa to 68% in Nouna) were the adolescents’ most commonly used modes of communication (Supplemental Table 1). Many adolescents increased their use of media such as television, movies, the internet, or social media, ranging from 24% in Kersa to 76% in Lagos (Supplemental Figure 4). Among those with some media consumption, the median leisure time spent on media consumption ranged from 2 hours per day (Q1: 1; Q3: 3) in Nouna to 5 hours per day (Q1: 3; Q3: 8) in Lagos (Supplemental Table 1).

### Sleep and nutrition before and during the COVID-19 pandemic.

The average duration of sleep before the COVID-19 pandemic ranged from 7.2 (SD: 1.5) hours per night in Lagos to 8.5 (SD: 1.6) hours per night in Ouagadougou (Supplemental Table 2). The average duration of sleep during the COVID-19 pandemic ranged from 7.6 (SD: 1.6) hours per night in Nouna to 9.3 (SD: 2.8) hours per night in Addis Ababa. Around half of the adolescents in Ouagadougou, Kersa, and both sites in Nigeria experienced no change in sleep duration after the pandemic (ranging from 45% in Lagos to 51% in Ouagadougou), whereas this proportion was higher (68%) in Nouna. In Addis Ababa, an increase in sleep duration was more common (49%).

**Figure 4.**
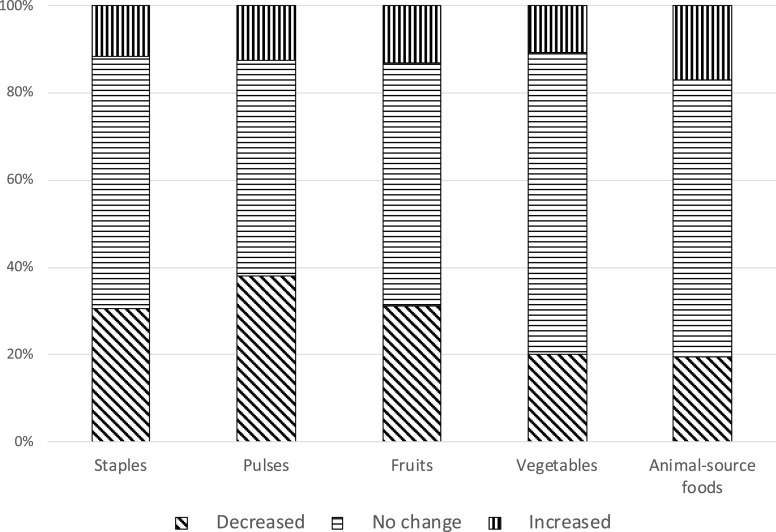
Change in the consumption of major food groups, including staples, pulses, fruits, vegetables, and animal-source foods, during the COVID-19 pandemic compared with before the pandemic among adolescents in a phone-based survey across six sites of three sub-Saharan African countries, 2020. The percentages correspond to the total sample with the six sites combined.

A decrease in food consumption was common across sites (Figure 4 and Supplemental Table 2), including decreases in the consumption of staples (ranging from 9% in Addis Ababa to 54% in Ibadan), pulses (ranging from 29% in Ouagadougou to 49% in Ibadan), fruits (ranging from 18% in Kersa to 41% in Ibadan), vegetables (ranging from 3% in Addis Ababa to 34% in Nouna), and animal-source foods (ranging from 11% in Kersa to 29% in Nouna).

### Impacts of the COVID-19 pandemic on mental health.

**Figure 5.**
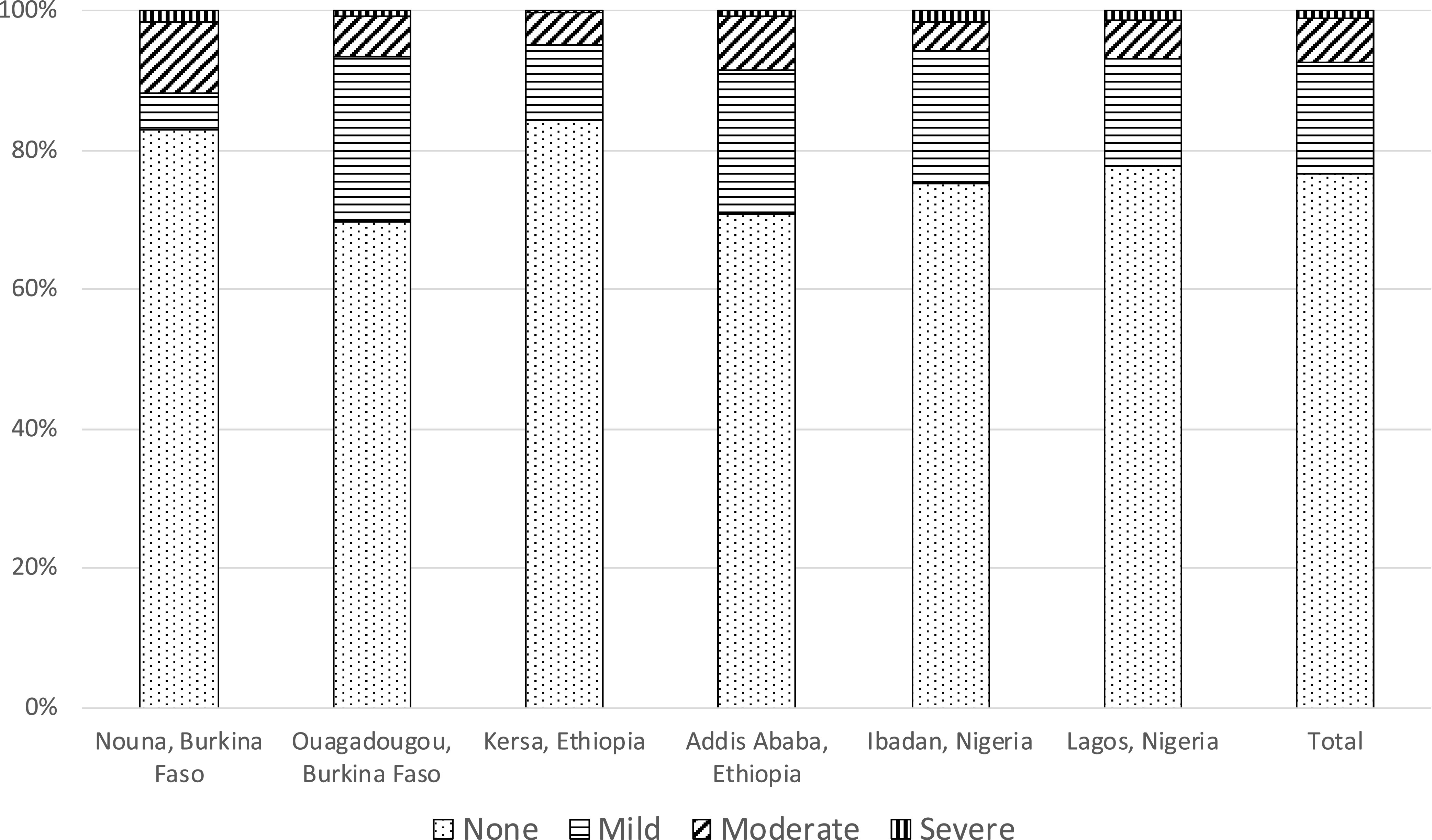
Levels of psychological distress during the COVID-19 pandemic among adolescents in a phone-based survey across six sites of three sub-Saharan African countries, 2020. Psychological distress was measured using the four-item Patient Health Questionnaire for Depression and Anxiety Scale. Each item a numeric score of 0, 1, 2, and 3, and the total score was computed by adding up the scores of the four items and had a range of 0–12. The total score of psychological distress was categorized into none (total score: 0–2), mild (total score: 3–5), moderate (total score: 6–8), and severe (total score: 9–12). Information was missing for two participants in Nouna, four participants in Ouagadougou, two participants in Kersa, five participants in Addis Ababa, 22 participants in Ibadan, and 19 participants in Lagos.

Most of the adolescents did not experience, or experienced less than half of the time, the four types of psychological distress included in the PHQ-4 (Supplemental Table 3). The median scores for psychological distress were 1 (out of 12) for Ouagadougou and Kersa and 0 for all other sites. The prevalence of moderate to severe psychological distress ranged from 4.8% in Kersa to 11.9% in Nouna (Figure 5). The median scores for anxiety and depression subscales were 0 in all sites except in Kersa (where the median was 1). A high score for the anxiety subscale was seen in around 10% of the adolescents, ranging from 7% in Ibadan to 14% in Nouna. A high score for the depression subscale was seen among 1% of the adolescents in Addis Ababa and 17% in Kersa, with other sites in between (Supplemental Table 3).

## DISCUSSION

This study examined adolescents’ knowledge, perceptions, and practices related to COVID-19 and the impacts of the COVID-19 pandemic on various aspects of adolescent lives in three countries in SSA. We found that adolescents’ knowledge of COVID-19 symptoms, transmission, and prevention was limited, especially in rural settings. We also found a major impact of the COVID-19 pandemic on adolescents’ access to education and evidence of reduced food intake likely resulting from the pandemic. This study is one of the first multi-country efforts to understand the impacts of the COVID-19 crisis on the lives of adolescents in SSA.

As COVID-19 continues to spread across the world, countries are taking unprecedented measures to mitigate the human and economic toll of the disease. Most countries in SSA have undertaken sweeping measures to limit transmissions, such as stay-at-home orders and physical distancing mandates.^1,21^ Although such measures are thought to have lowered the reproduction rate of the virus in sub-Saharan African countries,^21^ they also present unique direct and indirect consequences to the health and education systems, which were constrained even before the pandemic. The inadequate knowledge regarding the symptoms, transmission routes, and prevention strategies of COVID-19 among the adolescents in our study is concerning. A recent cross-sectional survey in Kampala, Uganda, also reported low uptake of COVID-19 prevention measures despite a high level of awareness of prevention measures.^14^ Although the COVID-19 symptoms are milder and the case fatality rate is lower among adolescents compared with older age groups,^6^ adolescents may still transmit the virus to vulnerable family or community members,^22^ and some adolescents do develop severe complications from COVID-19 infection.^23–25^ Therefore, urgent actions are needed to disseminate accurate information on COVID-19 to adolescents regarding the prevention of COVID-19 transmission in SSA, particularly in rural settings where awareness of the virus appears low.

Although adolescents have thus far been spared of the most severe direct health effects of the COVID-19 disease compared with other population groups, adolescents have unique education, developmental, and health needs, many of which may be hampered by the public health measures in place to combat the virus. One major impact of the pandemic on adolescents is the exacerbated inequities in education.^26^ The nationwide closures of educational establishments severely disrupt education for adolescents. Our study shows that the schools in which most interviewed adolescents attended were closed in response to the pandemic. Even in countries where alternative education channels such as radio, television, or online education programs were available during the COVID-19 crisis, they were not accessible or affordable to adolescents in poverty or remote areas.^27^ Our results show that online education was generally uncommon and particularly rare in rural or less developed settings. As a result, the school closures leave a large proportion of school-going adolescents with disrupted or suboptimal education and deprive many entirely of any education. Over half of the adolescents in most sites in our study self-reported to have less ability to continue learning during the pandemic. Therefore, in countries where primary and secondary schools have remained closed for a prolonged duration, rapid and adaptive actions need to be taken to address access to and the quality of remote learning opportunities for adolescents.^11^

In addition to the direct loss of formal education, the critical social protective functions of schools are lost with school closure, which may result in increased child labor, early marriages, adolescent pregnancies, transactional sex, gender-based violence, mental health issues, or exposures to risky behaviors such as tobacco smoking and drug use.^13,28–33^ Recently, a survey in Kampala, Uganda, reported that COVID-19 lockdowns were associated with a higher prevalence of unhealthy behaviors such as tobacco smoking/chewing, alcohol drinking, and substance use (such as marijuana).^14^ Due to the increased risks of such issues and the direct deprivation of educational opportunities, many adolescents in SSA may not go back to school even after schools reopen after a crisis like the COVID-19 pandemic.^29^ In the current study (except in Nigeria), around half of the adolescents perceived it very difficult to catch up on their education after the pandemic, a large proportion of whom may choose not to continue their education.

A rapid systematic review, based on studies mostly from high-income countries, suggests that children and adolescents may become more susceptible to depression and anxiety during and after enforced isolation due to the COVID-19 pandemic.^34^ The prevalences of depression, anxiety, and moderate to severe psychological distress were generally low across sites in our study. Our results are in line with a recent survey in Uganda, which reports that only 1.2% of adolescent boys or young men contemplated committing suicide due to COVID-19.^14^ Additional studies are needed to assess the long-term adverse consequences of the COVID-19 pandemic on the mental well-being of adolescents in SSA.

With the COVID-19 pandemic imposing devastating impacts on people’s livelihoods,^11^ 265 million people around the world were pushed into acute food insecurity in 2020, an increase of 130 million from 2019.^35^ In Zimbabwe, for example, the COVID-19 pandemic and the resultant lockdown measures have been associated with increased food prices, decreased dietary diversity, and lower levels of physical activity among adults.^36^ In Uganda, 62% of adolescent boys and young men found it difficult to afford a diverse and balanced diet during the COVID-19 pandemic.^14^ Adolescents have specific nutritional needs, and ensuring adequate diet and nutrition for adolescents is crucial for their health, development, and education.^37^ Rising food insecurity may exacerbate the high burden of undernutrition among adolescents in SSA.^38,39^ As a result of the COVID-19 pandemic and school closures in SSA, existing school nutrition programs, such as school feeding, that are prevalent in many countries^40^ have been disrupted.^41^ We report a potential reduction of adolescents' consumption of major food groups during the COVID-19 pandemic, particularly the reduced intake of staples, pulses, and fruits. Further studies in SSA are needed to understand the disruptive impacts of COVID-19 on school nutrition programs and the long-term effects of the pandemic on the diet and nutrition of adolescents.

The major strengths of this study are the inclusion of multiple sites in SSA, the use of novel CATI methodology to enable remote data collection, and the coverage of multiple relevant domains of the adolescent experience. This study also has some limitations. First, the study sites were selected opportunistically based on existing collaborations and infrastructure, and the adolescents at each site were not selected probabilistically.^15^ As a result, the sites included in each country are not representative of the national population. However, we intended to improve the generalizability of the results by including one rural site and one urban site in three countries spread across SSA. Therefore, we believe that the cross-country comparison of the results can nevertheless provide insights into the impacts of the COVID-19 on the adolescent experience in these and similar contexts. Second, due to the nature of the CATI methodology, we could not include adolescents whose households did not own a phone or who were not reachable by phone. However, recent evidence suggests that mobile phones are becoming ubiquitous in low-resource settings, with SSA seeing exponential growth in mobile phone use, including among youth.^42^ Third, information on dietary intake, physical activity, and health behaviors related to COVID-19 is self-reported by adolescents and may be subject to recall error or social desirability bias.^43^ Fourth, some variations in sampling frames and sampling strategies between sites may complicate the cross-site comparisons, as discussed in detail elsewhere.^15^ Fifth, due to the cross-sectional nature of the survey, we were not able to examine the more long-term impacts of the pandemic on adolescents, which we aim to pursue in a future effort by continuing this longitudinal survey. Last but not least, with the wide spectrum of domains covered, we did not specifically examine the individual- and country-level determinants for each domain, which is nevertheless essential for the designing of potential interventions and will be included in our future work.

Adolescents in SSA have historically been neglected regarding health and nutrition programming, with few efforts specifically targeting this critical life stage,^44^ and the ongoing COVID-19 crisis may exacerbate this trend. Children and adolescents are among the groups that will benefit the most from the recovery from the COVID-19 pandemic.^45^ As previously suggested,^45^ resource-constrained settings should move away from interventions that target only one outcome at a time but should instead search for actions that can improve multiple outcomes simultaneously (i.e., “accelerators”).^45^ To efficiently mitigate the impacts of the COVID-19 pandemic on adolescent education and nutrition, “accelerating” interventions should be evaluated, such as conditional cash transfers that households can use to access television or the internet for remote learning or purchase diverse and nutritious foods.

In conclusion, we find inadequate knowledge of COVID-19 among adolescents in SSA and considerable impacts of the COVID-19 pandemic on various aspects of adolescent experience, particularly the loss of education and decreased food consumption. This study serves as an initial evidence base for policymakers to design and implement interventions specifically targeted toward adolescents in SSA. Urgent actions should be taken at the national level to address the knowledge gap of COVID-19 and the unmet educational and nutritional needs of adolescents in SSA. Longitudinal studies are needed to understand the long-term impacts of the pandemic during the critically important life stage of adolescence.
